# Safety of PSMA radioligand therapy in mCRPC patients with preexisting moderate to severe thrombocytopenia

**DOI:** 10.1007/s00259-024-07006-z

**Published:** 2024-12-03

**Authors:** Moritz B. Bastian, Maike Sieben, Arne Blickle, Caroline Burgard, Tilman Speicher, Mark Bartholomä, Andrea Schaefer-Schuler, Stephan Maus, Samer Ezziddin, Florian Rosar

**Affiliations:** Department of Nuclear Medicine, Saarland University, Kirrberger Str., Geb. 50, 66421 Homburg, Germany

**Keywords:** Thrombocytopenia, PSMA, Radioligand therapy, mCRPC, Safety

## Abstract

**Purpose:**

Aim of this study was to analyze the safety of prostate-specific membrane antigen radioligand therapy (PSMA-RLT) in patients with metastatic castration-resistant prostate cancer (mCRPC) with preexisting moderate to severe thrombocytopenia (CTCAE ≥ 2).

**Materials and methods:**

Seventeen mCRPC patients with preexisting thrombocytopenia (platelet count < 75 × 10^9^/L) were included in this study. Patients received a median of 3 cycles of [^177^Lu]Lu-PSMA-617 (range 1–6). The course of platelet cell count was closely monitored within and after the PSMA-RLT and analyzed statistically and according to CTCAE.

**Results:**

No significant difference in platelet counts was observed between baseline and follow-up after each PSMA-RLT cycle: first cycle (54.18 ± 16.07 at baseline vs. 59.65 ± 39.16 at follow up [in × 10^9^/L], *p=*  0.834), second cycle (58.56 ± 16.43 vs. 107.1 ± 56.44, *p* = 0.203), and third cycle (60.38 ± 16.57 vs. 132.1 ± 80.43, *p* = 0.148), respectively. Similarly, baseline and end of treatment values, irrespective of the number of administered cycles, did not reveal a significant difference (54.18 ± 16.07 vs. 72.06 ± 71.9, *p* = 0.741). After the end of therapy, irrespective of the number of administered cycles, 29.4% of patients remained stable in terms of CTCAE scoring, 41.2% changed to a higher score and 29.4% improved to a lower score. We observed no critical bleeding events due to thrombocytopenia.

**Conclusion:**

Despite the common consideration of marked preexisting thrombocytopenia as a contraindication for RLT, this study indicates feasibility of PSMA-RLT in patients with preexisting thrombocytopenia of grade ≥ 2, as in our preliminary experience, there was no RLT-induced significant deterioration of platelet cell count. Thus, patients with thrombocytopenia should not be categorically excluded from receiving PSMA-RLT.

**Supplementary Information:**

The online version contains supplementary material available at 10.1007/s00259-024-07006-z.

## Introduction

Prostate cancer (PC) is currently listed as the second most abundant malignancy on a global scale [1]. PC frequently progresses into metastatic castration-resistant prostate cancer (mCRPC), which is associated with a poor prognosis [2–4]. Besides novel androgen axis drugs (NAAD) [5, 6], taxane based chemotherapy [7, 8], ^223^Ra treatment [9] or PARP-inhibitors [10, 11]), radioligand therapy (RLT) targeting the prostate-specific membrane antigen (PSMA), which is overexpressed on mCRPC cells [12, 13], is a promising treatment option for mCRPC [14–20]. PSMA-RLT has revealed a favorable side effect profile, however a limited number of hemotoxicities occurred, e.g. 17% of patients were exhibiting thrombocytopenia during the VISION-trial [21]. Accordingly, the joint EANM/SNMMI procedure guideline acknowledges myelosuppression, i.e. preexisting thrombocytopenia, as a contraindication for [^177^Lu]Lu-PSMA-617 RLT [22]. However, there is limited data on this topic, while clinical experience suggests that pre-existing thrombocytopenia may not necessarily disqualify patients from PSMA-RLT. This study aims to analyze the safety of PSMA-RLT in patients with preexisting thrombocytopenia.

## Materials and methods

In total, n = 17 mCRPC patients with pre-existing thrombocytopenia, receiving RLT were included in this study. Thrombocytopenia was defined as platelets count < 75 × 10^9^/L, equaling a score ≥ 2, according to the ‘*common terminology criteria of adverse events*’ (CTCAE v5.0). The patients were all in a very advanced stage of mCRPC and had exhausted standard treatments, where PSMA-RLT remained the last therapeutic option. The potential effectiveness of the radioligand modality and the clinical need in the individual situation were critical factors in our decision-making process in the presence of significant thrombocytopenia. All patients received ADT and 14/17 (82.4%) NAAD prior and/or ongoing. In total, 14/17 (82.4%) were previously treated with chemotherapy (ending median 7 months, range 2–24 months prior) and 5/17 (29.4%) with bone-seeking ^223^Ra (ending median 4 months, range 1–6 months prior). Details of patient characteristics are summarized in Table 1.

**Table 1:** Patient characteristics

Patient characteristics	Value
Age	
Median in [years], (range)	66 (50–81)
Age ≥ 65 years, *n* (%)	10 (58.8)
Age < 65 years, *n* (%)	7 (41.2)
ALP, in [U/L]	
Median (range)	183 (44–971)
Hemoglobin, in [g/dL]	
Median (range)	9 (5–13.3)
< 13.5 g/dL, *n* (%)	17 (100)
ECOG performance status, *n* (%)	
0	1 (5.9)
1	5 (29.4)
≥ 2	11 (64.7)
Prior therapies, *n* (%)	
Prostatectomy	7 (41.2)
Radiation	9 (52.9)
ADT	17 (100)
NAAD	14 (82.4)
-Abiraterone	12 (70.6)
-Enzalutamide	13 (76.5)
-Abiraterone and Enzalutamide	9 (52.9)
Chemotherapy	14 (82.4)
-Docetaxel	14 (82.4)
-2nd line Cabazitaxel	7 (41.2)
[^223^Ra]Ra-dichloride	5 (29.4)
Other	9 (52.9)
PSA at baseline, in [ng/mL]	
Median (range)	852 (0.19–4832)
Sites of metastases, *n* (%)	
Bone	16 (94.1%)
-with diffuse bone marrow involvement	6 (35.3%)
Lymph node	10 (58.8%)
Liver	5 (29.4%)
Lung	1 (5.9%)

*ADT* androgen deprivation therapy, *ALP* alkaline phosphatase, *ECOG* Eastern Cooperative Oncology Group, *NAAD* novel androgen axis drugs, *PSA* prostate specific antigen

Following the German Pharmaceutical act §13 (2b), PSMA-RLT was performed on a compassionate use basis. PSMA-RLT was performed during an inpatient stay at our institution. At our center, patients with platelet counts below 75 × 10^9^/L may still be given the chance of receiving PSMA-RLT in the context of missing alternative systemic treatment options. Each case is discussed on an individual basis in our multidisciplinary tumor board, taking into account the severity of blood count abnormalities or bone marrow dysfunction, clinical condition, and treatment pressure (clinical pressure to achieve remission in the view of the disease burden and dynamics). The idea behind offering the RLT modality in this specific setting on an individual basis was to provide potentially life-prolonging treatment to patients who might otherwise have no remaining viable therapeutic options. Informed consent was obtained from all patients after discussing the potential risks and benefits of the therapy, ensuring that they were fully aware of the implications of undergoing treatment in the setting of impaired bone marrow function. The radiolabeling and the quality control were performed according to the established standard procedures [23, 24]. Patients received a median of 3 cycles PSMA-RLT (range 1 – 6) with a median time interval of 6 weeks between consecutive cycles of [^177^Lu]Lu-PSMA-617. The mean administered activity of [^177^Lu]Lu-PSMA-617 per cycle was 6.9 ± 1.7 GBq (2.7–11.0 GBq) and the mean cumulative activity was 17.5 ± 10.8 GBq (2.7–42.7 GBq), respectively. The guideline recommended ^177^Lu activities for non-compromised patients [22] were considered as basis, and further personalized dosing was implemented in attempt to optimize therapeutic outcomes while minimizing risks. The administered activities were adjusted individually, based on the characteristics of each patient, considering tumor burden, therapy pressure, diffuse involvement of bone marrow, course of disease, general patient condition, and functional blood parameters as previously introduced by Khreish et al. [14]. In addition, 4 patients being part of the analysis received 1–3 [^225^Ac]Ac-PSMA-617- augmented cycles (in total 6 cycles) within [^177^Lu]Lu-PSMA-617 RLT, with a mean cumulative [^225^Ac]Ac-PSMA-617 activity of 6.9 ± 6.5 MBq (range: 2.2–16.3 MBq) and a mean activity of 4.6 ± 1.9 MBq (range: 2.2–7.6 MBq) per cycle. Within the augmented therapy the mean [^177^Lu]Lu-PSMA-617 activity per cycle was 7.0 ± 0.9 GBq (range 5.8–8.3 GBq). Detailed information on applied activities is compiled in the supplementary material (Table S1).

The course of platelet cell count was closely monitored within and after the PSMA-RLT and analyzed statistically and according to CTCAE, with baseline laboratory tests < 24 h before administration of the first PSMA-RLT cycle and subsequent frequent blood sampling either in-house or at the referring physician's office (general practitioner, urologist or oncologist). For statistical analysis, descriptive analysis and Wilcoxon matched pairs signed rank test was performed using Prism 8 (GraphPad Software, San Diego, CA, USA), to evaluate possible differences in platelet counts between baseline and follow-up examinations. A *p*-value < 0.05 was defined as statistically significant.

## Results

**Fig. 1:**
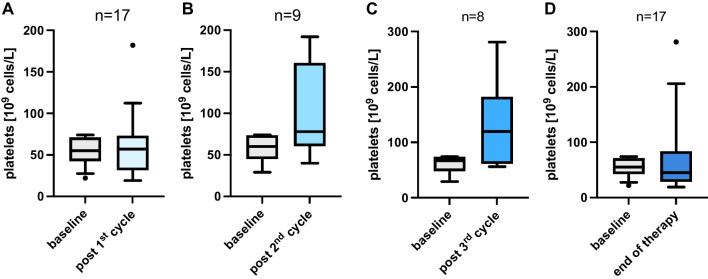
Box plots presenting a comparison of the platelet count at baseline and after (**A**) first cycle (**B**) second cycle, (**C**) third cycle, and (**D**) end of PSMA-RLT

Comparing the platelet cell count of baseline to the follow up values after the first RLT cycle, no significant difference was found (54.18 ± 16.07 vs. 59.65 ± 39.16 [in × 10^9^/L], *p* = 0.834, n = 17, Fig. 1A). Similarly, in patients receiving at least two cycles no significant difference was observed contrasting baseline and follow up values after the second cycle (58.56 ± 16.43 vs. 107.1 ± 56.44, *p* = 0.203, n = 9, Fig. 1B). Neither did the comparison of baseline and follow up values after the third treatment cycle show any significant difference (60.38 ± 16.57 vs. 132.1 ± 80.43, *p* = 0.148, n = 8, Fig. 1C). Analogously, baseline and end of treatment values, irrespective of the number of administered cycles, did not reveal a significant difference (54.18 ± 16.07 vs. 72.06 ± 71.9, *p* = 0.741, n = 17, Fig. 1D). In terms of PSA response, the mean best response of the cohort was −21.4 ± 64.1%.

**Fig. 2:**
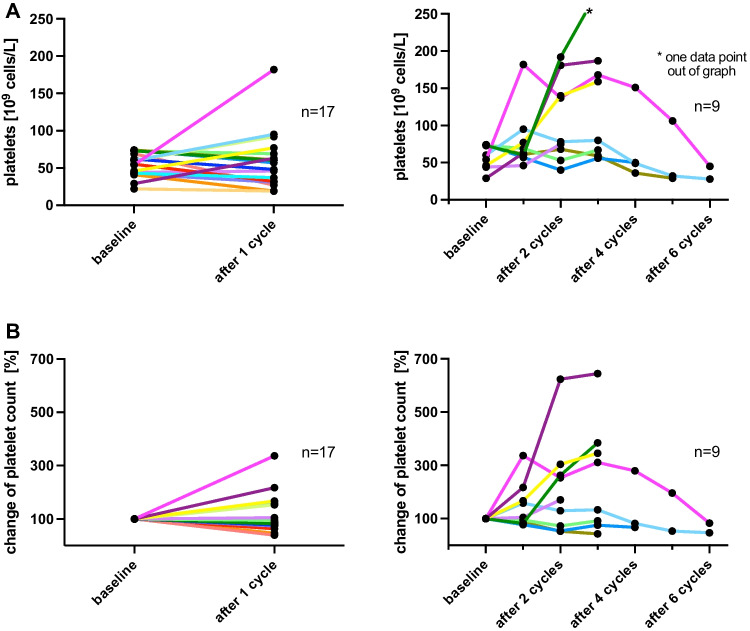
**A** Left: absolute platelet cell count of all patients at baseline and after one cycle of RLT. Right: course of absolute platelet cell count of patients who received two or more cycles of RLT. **B** Left: relative platelet cell count of all patients at baseline and after one cycle of RLT. Right: relative platelet cell count of patients who received two or more cycles of RLT

**Fig. 3:**
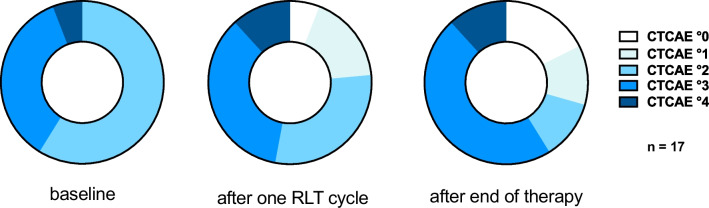
CTCAE scores for thrombocytopenia at baseline, after the first cycle and after the end of [^177^Lu]Lu-PSMA-617 RLT

The individual analysis of baseline platelet counts juxtaposed with those after the initial cycle and in course of [^177^Lu]Lu-PSMA-617 RLT regime reveals a consistent stability in platelet counts for the majority of patients. In certain cases, a noticeable increase was observed, whilst platelet counts decreased in a small number of cases as well (Fig. 2). Following the common terminology criteria for adverse events (CTCAE v5.0), 8/17 patients (47.1%) maintained the same grade of thrombocytopenia after one cycle of [^177^Lu]Lu-PSMA-617 administration, 5/17 patients (29.4%) showed an improvement resulting in a lower CTCAE score, while 4/17 patients (23.5%) progressed to a higher grade (Fig. 3). After the end of therapy, irrespective of the number of administered cycles, 5/17 patients (29.4%) remained stable in terms of CTCAE scoring, 7/17 patients (41.2%) changed to a higher score and 5/17 patients (29.4%) improved to a lower CTCAE score during RLT (Fig. 3). No critical event of spontaneous bleeding occurred.

Considering further hematological parameters: pre RLT hemoglobin level was mean 9.72 ± 1.92 g/dL (range 5.0 – 13.3), and post RLT 9.18 ± 1.93 g/dL (range 5.7–13.1 g/dL), respectively. In terms of CTCAE grading all 17 patients showed anemia pre RLT (6 patients with grade 1, 10 with grade 2 and 1 with grade 3). Four patients experienced a deterioration in their CTCAE grade, while the rest remained stable. The proportions of CTCAE grades for anemia pre- and post-RLT are summarized in the supplementary material (Table S2). In terms of leukocytes counts, the mean value pre RLT was 4.54 ± 2.69 × 10^9^/L (range 2 – 13 × 10^9^/L), and 3.45 ± 1.69 × 10^9^/L (range 1–8.7 × 10^9^/L) post RLT, respectively. 7 patients had leukocytopenia prior to RLT and 11 patients had leukocytopenia post RLT. The proportions of CTCAE grades for leukopenia pre- and post-RLT are summarized in the supplementary material (Table S3). In total 7 patients had pancytopenia prior to PSMA-RLT and 11 patients had pancytopenia post PSMA-RLT. Two patients discontinued treatment due to pancytopenia and deterioration in the patient's general condition.

**Fig. 4:**
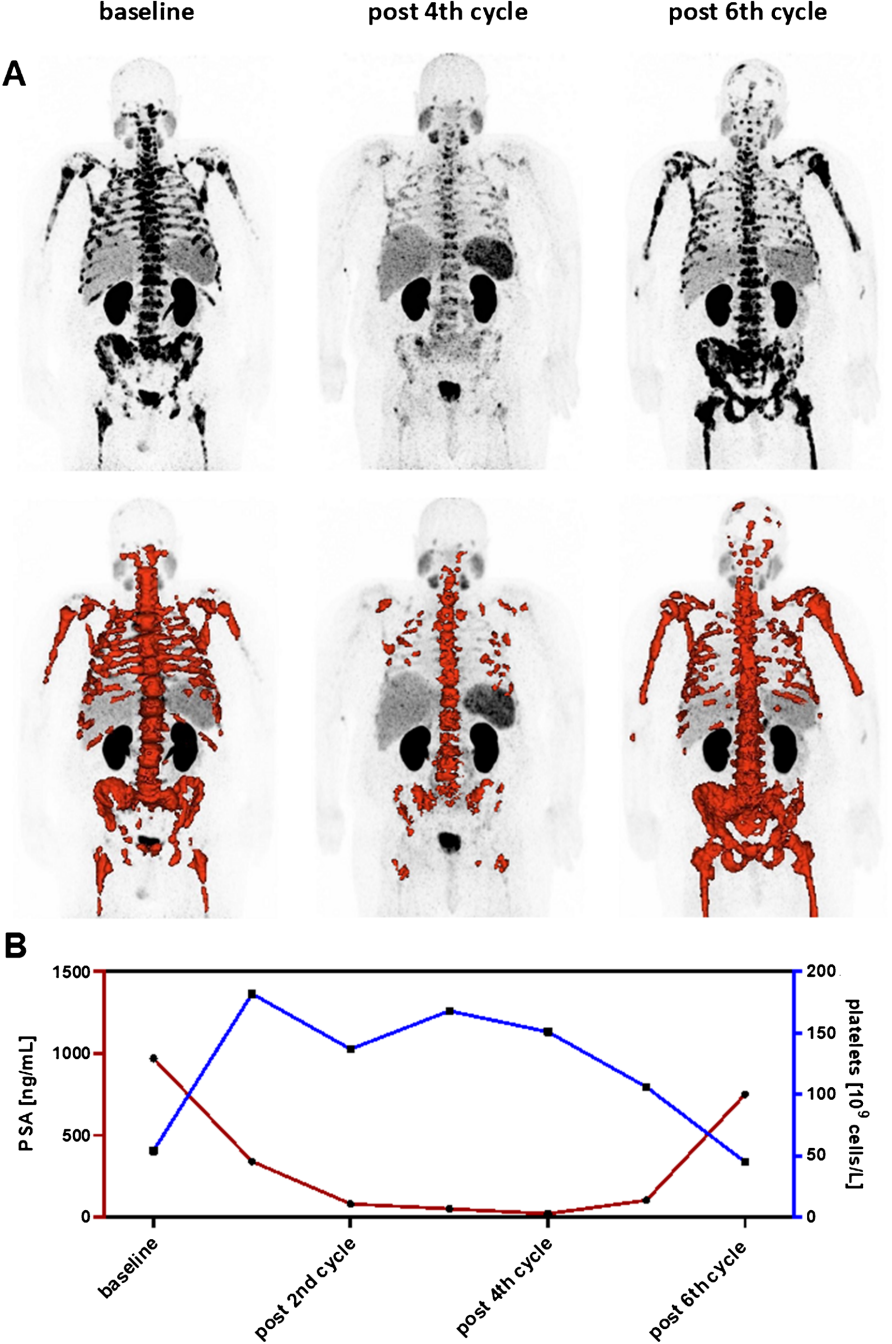
Exemplary patient with initial thrombocytopenia treated with 6 cycles of [^177^Lu]Lu-PSMA-617 RLT. **A** Maximum intensity projections of [^68^ Ga]Ga-PSMA-11 PET imaging at baseline, after 4 cycles, and after 6 cycles of [^177^Lu]Lu-PSMA-617 RLT. Total tumor burden highlighted in red. **B** Course of platelet cell counts and PSA levels during treatment

Figure 4 depicts an exemplary patient who received six cycles of [^177^Lu]Lu-PSMA-617. While prostate specific antigen (PSA) and tumor burden in molecular imaging clearly decreased between baseline and 4th cycle, a simultaneous rise of platelet count that remained at a relatively high level was noted. However, when progression of the tumor was observed, the platelet cell count decreased again.

## Discussion

While a low cell count of platelets is often considered as contraindication for [^177^Lu]Lu-PSMA-617 RLT in clinical practice, the preliminary results of this study strongly indicate that a preexisting thrombocytopenia is not necessarily an exclusion criterion for this kind of treatment.

While most studies have analyzed RLT side effects in patients with platelet counts within the normal range, to the best of our knowledge, this is the first study to investigate RLT-related adverse events in cohort with preexisting thrombocytopenia of grade ≥ 2. The adverse event of thrombocytopenia is reported in [^177^Lu]Lu-PSMA-617 RLT by several studies, for example the VISION-trial by Sartor et. al reported a thrombocytopenia occurrence of 17.2% [20], the REALITY-study by Khreish et. al stated an occurrence of 22.4% [14], and the TheraP-trial by Hofmann et al. [18] stated an occurrence rate of 29%, respectively. Consequently, preexisting thrombocytopenia is regarded as a risk factor, potentially leading to the exclusion of patients from RLT. In accordance, the joint guidelines from the European Association of Nuclear Medicine (EANM) and the Society of Nuclear Medicine and Molecular Imaging (SNMMI) for [^177^Lu]Lu-PSMA RLT state that a platelet count of less than 75 × 10^9^/L is a relative contraindication for treatment [22].

By analyzing a cohort of patients with preexisting thrombocytopenia undergoing RLT, this study demonstrated that the platelet cell count of these individuals did not decrease significantly. Instead, it remained stable for the majority and even increased for some patients, and only decreased for a few patients, with only two patients discontinuing treatment due to pancytopenia and a severe deterioration in the patient's general condition. Frequently, extensive changes in platelet count (increase and decrease) were most likely attributed to, the regression or progression of the tumor disease (e.g. Fig. 4). Moreover, in patients with (diffuse) bone metastasis, the involvement of the bone marrow certainly negatively impacts hematopoiesis, potentially worsening thrombocytopenia. The observed post-therapy thrombocytopenia in some cases could also be related to disease progression and corresponding affection of bone marrow function, accompanied by deterioration of patient condition.

Given the limitation of a rather small sample size, the presented data should be regarded as preliminary findings on a crucial subject. In addition, individual activities and no fixed activity protocol was used potentially influencing outcome and side effects.

The appropriate applied activity of ^177^Lu should be investigated in this setting and an adapted protocol for these patients may be defined in the future. Furthermore, the majority of patients received only limited number of cycles and the study focuses on short-term safety. Long-term safety and survival follow-up should be evaluated in subsequent studies, ideally employing larger cohorts and a prospective study design.

Another potential limitation of this study is the time interval between prior chemotherapy or ^223^Ra therapy and the initiation of PSMA-RLT (median 7 and 4 months, at least 1 and 2 months, respectively). It should be noted that these therapies can have transient effects on bone marrow function, allowing platelet counts to recover which could bias the results. Moreover, 4 patients included in the analysis received alpha-augmented RLT with [^225^Ac]Ac-PSMA-617 which may impact the results. However, based on the experience from these 4 cases, even in the presence of thrombocytopenia, this combination seems not to be accompanied with additional negative thrombopoietic effects. Alternative approaches, such as utilizing solely an alpha-emitter like ^225^Ac with a shorter particle range, may more effectively spare healthy bone marrow, warrant further evaluation.

## Conclusion

Despite the common consideration of marked preexisting thrombocytopenia as a contraindication for RLT, this study indicates feasibility of PSMA-RLT in patients with preexisting thrombocytopenia of grade ≥ 2, as in our preliminary experience, there was no RLT-induced significant deterioration of platelet cell count. Thus, patients with thrombocytopenia should not be categorically excluded from receiving PSMA-RLT.

## Supplementary Information

Below is the link to the electronic supplementary material.

Supplementary file1 (DOCX 17 kb)

## Data Availability

The datasets generated during and analyzed during the current study are available from the corresponding author on reasonable request.
