# Supplementary material for: Safety of PSMA radioligand therapy in mCRPC patients with preexisting moderate to severe thrombocytopenia
Source: Eur J Nucl Med Mol Imaging. 2024 Dec 3;52(4):1271–7. doi: 10.1007/s00259-024-07006-z (PMC11839894; doi:10.1007/s00259-024-07006-z)
Supplement: Supplementary file 1 — Supplementary file1 (DOCX 17 kb) [file 259_2024_7006_MOESM1_ESM.docx]

| Patient # | Administered Activities of ^177^Lu [GBq] (+^255^Ac [MBq]) per cycle | Characteristics | Δ PSA[%] | Response (PCWG3) |
| --- | --- | --- | --- | --- |
| 1 | 7.3 /7.0 / 6.5 | BP | -94.5 | PR |
| 2 | 10.2 | BM, BP, TB, TP, VM | +40.6 | PD |
| 3 | 6.0 / 7.4 | BP, VM | +80.8 | PD |
| 4 | 8.4 | BP, TP, VM | +2.2 | SD |
| 5 | 5.7 / 5.6 /5.8 | BP, RPC | -99.0 | PR |
| 6 | 8.5 / 8.4 / 8.7 | BP, TB | -54.3 | PR |
| 7 | 5.8 (+7.6) / 7.4 (+4.1) / 7.2 (+4.6) | BP, TB | -59.2 | PR |
| 8 | 6.5 | BP, BM | +47.0 | PD |
| 9 | 8.8 / 6.8 / 7.4 | BP | -99.0 | PR |
| 10 | 8.2 / 7.0 | BP, BM, TP, VM | +75.7 | PD |
| 11 | 2.7 | BP, RPC, BM | -19.1 | SD |
| 12 | 5.7 | BP, RPC | +29.8 | PD |
| 13 | 7.2 / 7.0 / 3.6 / 5.7 / 5.2 | BP, VM | -95.8 | PR |
| 14 | 4.0 | BP, BC, BM | -11.6 | SD |
| 15 | 8.3 (+3.1) / 8.5 /6.9 | BP, TB | +25.9 | PD |
| 16 | 11.0 / 7.1 / 5.2 / 8.3 / 5.3 / 5.8 (+5.8) | BP, BM, TB, TP | -97.8 | PR |
| 17 | 9.5, 5.7, 7.1 (+2.2), 7.3 | BP, TP, TB | -52.9 | PR |

**Supplementary table S1**. Applied individual patient activities per cycle, PSA change in %, the response, and patient characteristics (TP (high clinical treatment pressure), BM (diffuse bone marrow involvement), TB (excessive tumor burden), BP (impaired functional blood parameters), RPC (markedly reduced patient condition), VM (visceral metastases)); progressive disease (PD); partial remission (PR); stable disease (SD).

**Supplementary table S2.** CTCAE v5.0 Anemia (LLN: 13.5 g/dL):

| CTCAE Grade | Pre-RLT | Post-RLT |
| --- | --- | --- |
| °0 | 0 | 0 |
| °1 | 6 | 5 |
| °2 | 10 | 10 |
| °3 | 1 | 2 |
| °4 | 0 | 0 |

**Supplementary table S3.** CTCAE v5.0 White blood cell decrease (LLN: 4.0 × 10^9^ L):

| CTCAE Grade | Pre-RLT | Post-RLT |
| --- | --- | --- |
| °0 | 10 | 6 |
| °1 | 2 | 6 |
| °2 | 5 | 2 |
| °3 | 0 | 3 |
| °4 | 0 | 0 |
